# Risk factors and clinical prediction formula for the evaluation of obstructive sleep apnea in Asian adults

**DOI:** 10.1371/journal.pone.0246399

**Published:** 2021-02-02

**Authors:** Do-Yang Park, Ji-Su Kim, Bumhee Park, Hyun Jun Kim

**Affiliations:** 1 Department of Otolaryngology, Ajou University School of Medicine, Suwon, Republic of Korea; 2 Sleep Center, Ajou University Hospital, Suwon, Republic of Korea; 3 Office of Biostatistics, Ajou Research Institute for Innovative Medicine, Ajou University Medical Center, Suwon, Republic of Korea; 4 Department of Biomedical Informatics, Ajou University School of Medicine, Suwon, Republic of Korea; University of Catania, ITALY

## Abstract

Obstructive sleep apnea is a highly prevalent cyclic repetitive hypoxia-normoxia respiratory sleep disorder characterized by intermittent upper-airway collapse. It is mainly diagnosed using in-laboratory polysomnography. However, the time-spatial constraints of this procedure limit its application. To overcome these limitations, there have been studies aiming to develop clinical prediction formulas for screening of obstructive sleep apnea using the risk factors for this disorder. However, the applicability of the formula is restricted by the group specific factors included in it. Therefore, we aimed to assess the risk factors for obstructive sleep apnea and develop clinical prediction formulas, which can be used in different situations, for screening and assessing this disorder. We enrolled 3,432 Asian adult participants with suspected obstructive sleep apnea who had successfully undergone in-laboratory polysomnography. All parameters were evaluated using correlation analysis and logistic regression. Among them, age, sex, hypertension, diabetes mellitus, anthropometric factors, Berlin questionnaire and Epworth Sleepiness Scale scores, and anatomical tonsil and tongue position were significantly associated with obstructive sleep apnea. To develop the clinical formulas for obstructive sleep apnea, the participants were divided into the development (n = 2,516) and validation cohorts (n = 916) based on the sleep laboratory visiting date. We developed and selected 13 formulas and divided them into those with and without physical examination based on the ease of application; subsequently, we selected suitable formulas based on the statistical analysis and clinical applicability (formula including physical exam: sensitivity, 0.776; specificity, 0.757; and AUC, 0.835; formula without physical exam: sensitivity, 0.749; specificity, 0.770; and AUC, 0.839). Analysis of the validation cohort with developed formulas showed that these models and formula had sufficient performance and goodness of fit of model. These tools can effectively utilize medical resources for obstructive sleep apnea screening in various situations.

## Introduction

Obstructive sleep apnea (OSA) is a sleep-related disorder characterized by repeated episodes of partial or complete upper airway obstruction during sleep. It has a reported prevalence of 3–9% in the general population [1]. OSA is associated with resistant hypertension (HTN) and cardiovascular disease; specifically, it is associated with an increased cardiovascular event rate, including myocardial infarction and stroke; atrial fibrillation; insulin resistance; increased cancer incidence and mortality; neurodegeneration; and hypoxic burden [2–7]. Based on the pathophysiology, there are different endotypes of OSA; moreover, drug therapy has been attempted [8]. Over the years, there have been increasing social costs of sleep disorders [9]. Specifically, there has been increasing social concern regarding healthy sleep due to traffic accidents caused by daytime sleepiness, which is a major sleep apnea symptom, and large-scale disasters caused by a lack of attention [10–12]. Consequently, sleep-related social policies have been adopted worldwide [13–15]. However, there are differences between the predicted prevalence and actual diagnosis rate, with some individuals remaining undiagnosed. In Western countries, up to 5% of the population has undiagnosed OSA syndrome (elevated apnea-hypopnea index [AHI] and symptoms) [16]. Notably, approximately 80% of men and 93% of women remain undiagnosed [17].

The gold standard for OSA diagnosis is in-laboratory polysomnography (PSG) [18, 19]. Typically, patients with suspected OSA initially visit out-patient sleep clinics. Subsequently, they undergo medical/physical examination and in-laboratory/out-center PSG based on their sleep specialist's prescription. During this process, medical experts decide on the PSG prescription for patients with suspected OSA, with their age, sex, weight, and hormonal change being possible sleep-related risk factors. Previously reported sleep-related risk factors with strong associations include obesity, male sex, old age, and menopause, while those with moderate associations include craniofacial/upper-airway abnormalities, smoking, alcohol drinking, nasal congestion, cardiovascular disease, and family history of sleep apnea [20].

Given the significant negative effects of OSA, proper diagnosis and treatment of patients with suspected OSA are important. However, the available diagnostic modality has several limitations, including being time-consuming, expensive, and unfit for specific situations. To overcome this, there have been various studies to analyze risk factors for OSA as a method of screening and develop clinical formulas for OSA screening based on these factors [21]. However, these previously developed formulas are restricted to target groups with the same characteristics as that of the enrolled participants; their applicability is also limited by the analyzed risk factors included in the formula, such as questionnaires, physical examination, radiologic factors. Therefore, to recommend PSG for patients with suspected OSA, we aimed to review various medical history data, including demographic, anthropometric, physical, and polysomnographic characteristics. Moreover, we aimed to analyze risk factors for OSA and develop and present various formulas that achieved a certain high statistical standard; the selected formulas can be modified according to various situations. We have also tried to verify and present one formula that requires a physical exam and one that does not.

## Materials and methods

### Study participants

**Fig 1:**
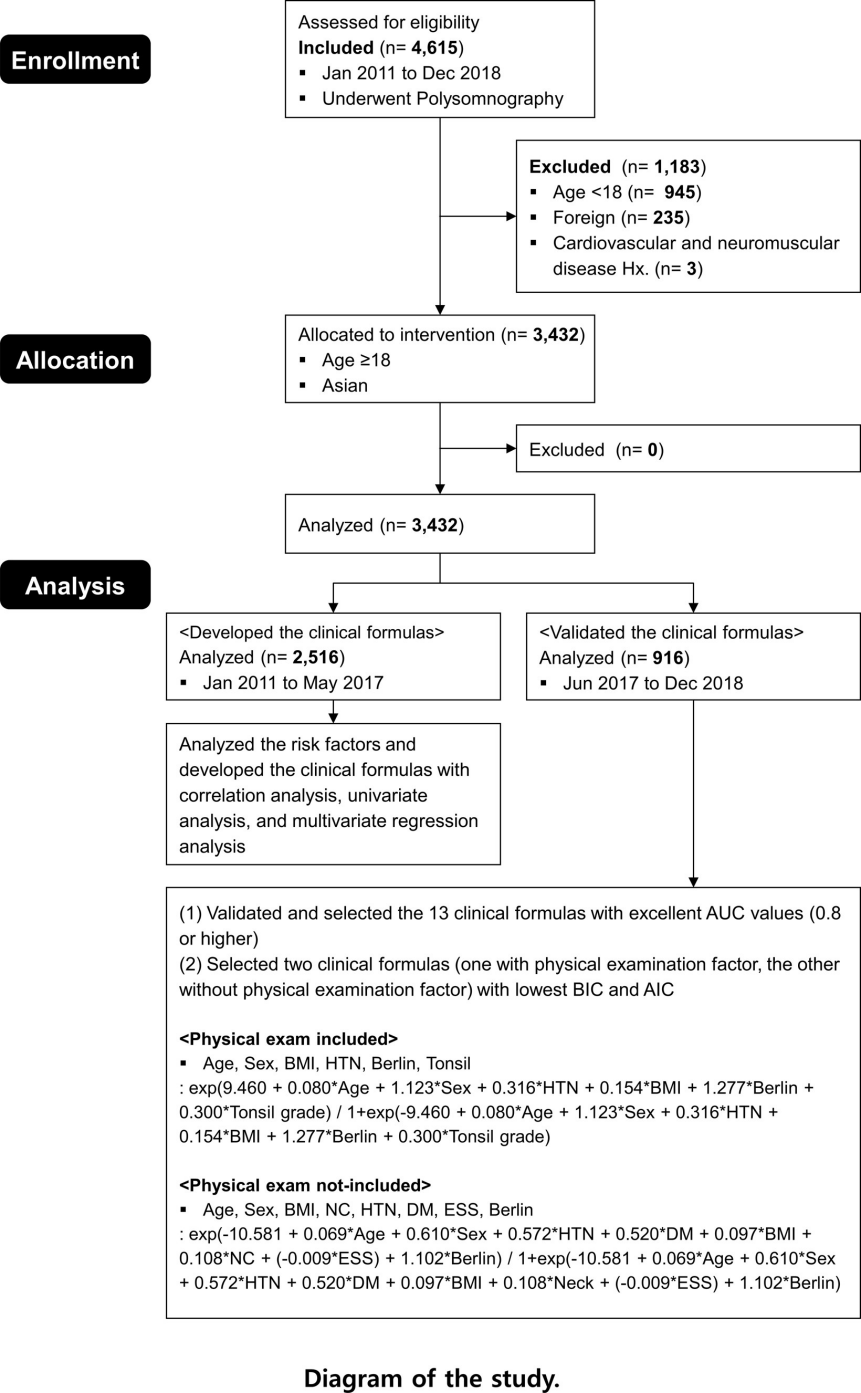
Schematic diagram of the study process.

Among the patients with suspected OSA from January 2011 to December 2018, we enrolled 4,615 patients who visited the hospital and had undergone a PSG test. We excluded 945 children aged < 18 years and 245 foreigners for racial uniformity of the study participants. Further, we excluded 3 participants with a severe disease history of cardiovascular and neuromuscular systems. Finally, 3,432 participants were enrolled for analysis. This study collected PSG; medical history; anthropometric data, which are known to affect OSA, as well as results of the sleep-related questionnaire and physical oropharynx examination. Using statistical methods, we confirmed normal distribution of our study dataset, which represented a larger population. According to the visit time, we divided the data of 3,432 participants into two groups; specifically, one for developing the clinical prediction formula (n = 2516; January 2011 to May 2017; development cohort) and the other for verifying the clinical formula (n = 916; June 2017 to December 2018; validation cohort). Using the 3,432 enrolled participants, we analyzed the risk factors for OSA. Subsequently, using the development cohort, we employed the identified risk factors to develop a clinical prediction formula for OSA and validated using the validation cohort (Fig 1).

### Ethical considerations

The study protocol was reviewed and approved by the Institutional Review Board of Ajou University Hospital (MED-MDB-19-252).

### Polysomnography (PSG)

PSG (Embla N7000, ResMed, Amsterdam, Netherlands) recording was performed using six electroencephalography channels (C3-A2, C4-A1, F3-A2, F4-A1, O1-A2, and O2-A1), two electrooculogram channels (ROC-A1 and LOC-A2), electromyogram and electrocardiogram leads, and pulse oximetry. Further, we employed an oronasal thermal airflow sensor, nasal pressure transducer, thoracic and abdominal respiratory supports using plethysmography belts, and body position sensors. Apnea was defined as a ≥ 90% reduction in the respiratory signal amplitude, as shown by the oronasal thermal airflow sensor or respiratory inductive plethysmography (RIP) sensor, compared with the baseline amplitude for > 10 s. Specifically, it was classified as obstructive, central, or mixed based on the presence or absence of respiratory efforts. Hypopnea was defined as a ≥ 30% reduction in the respiratory signal amplitude, as indicated by a nasal pressure airflow sensor or RIP sensor, compared with the baseline amplitude for > 10 s, with an accompanying decrease of ≥ 3% in SaO_2_ and arousal with associated events or a decrease of ≥ 4% in SaO_2_. The AHI was defined as the number of obstructive and/or mixed apneas as well as the number of hypopneas per hour of total sleep. Respiratory effort-related arousal (RERA) was scored in case there was a breath sequence lasting > 10 s involving increased respiratory effort, flattening of the inspiratory portion of the nasal pressure, or snoring, which led to arousal from sleep, with the breath sequence not meeting the criteria for apnea or hypopnea. The respiratory disturbance index (RDI) was calculated by dividing the total number of apneas, hypopneas, and RERAs by the total sleep time. Nadir oxygen saturation was defined as the lowest oxygen saturation measured using the pulse oximeter. All PSG data were manually scored by a sleep specialist based on the most recent American Academy of Sleep Medicine 2012 criteria [22].

### Assessment of demographic characteristics, anthropometric measurements, and physical examination

Medical records and self-reported questionnaires were used to obtain the medical history of HTN, diabetes mellitus (DM), and allergy. The presence of allergy was defined as a positive result in an allergy test, including radioallergosorbent test, multiple allergen simultaneous test, and skin prick test, in the medical records. A medical expert measured the weight; height; and neck, waist, and hip circumferences. BMI was calculated as weight in kilograms divided by height in meters squared (kg/m^2^), as recommended by the International Obesity Task Force and the World Health Organization Regional Office for the Western Pacific Region for Asian individuals [23]. After the participants had completed the questionnaire, they were physically examined by a sleep specialist. According to Friedman's clinical staging and modified Mallampati index for sleep-disordered breathing, tonsil was graded from I to IV; cases where the tonsil tissue was not visible were defined as I; cases where the tonsil was visible in the pillars as II; cases with tonsils outside the pillars as III; and cases where tonsils reached the midline as grade IV. Similarly, tongue position grading was performed as follows: I, if uvula and the entire tonsils/pillar were clearly visible; II, if most of the uvula was visible but tonsils or the pillar was invisible; III, if only the soft palate was partially visible; and IV, if only the hard palate was visible [24]. The uvula length was divided into stages I to III, which corresponded to short, moderate, and long, respectively. Moreover, the oropharyngeal width was divided into stages I to III, which corresponded to ‘no obstruction,’ ‘partial obstruction,’ and ‘complete obstruction’ between both lateral pharyngeal walls, respectively.

### Questionnaire for sleep quality evaluation

Subjective sleep quality evaluation was performed using the validated Korean version of the Pittsburgh Sleep Quality Index (PSQI) [25]. A global score of > 5 was indicative of poor sleep quality [26]. The validated Korean version of the ESS was used to evaluate excessive daytime sleepiness [27]. OSA symptoms and clinical predictors were evaluated using the validated Korean version of the Berlin questionnaire [28].

### Statistical analysis

Normally distributed continuous variables were presented as means and standard deviations while non-normally distributed variables were presented as medians and interquartile ranges. The normal distribution of variables was determined using the Kolmogorov-Smirnov test. Risk factors for OSA were determined using Pearson’s correlation analysis and univariate logistic regression. Multivariate logistic regressions were used to analyze the relationships between the risk factors and OSA. To analyze the explanatory power and verify the multivariate logistic regression model, we performed Cox & Snell R^2^, Nagelkerke R^2^ and Hosmer–Lemeshow goodness-of-fit tests. Further, we used receiver operating characteristic (ROC) curves to verify the clinical prediction formula. We used area under the curve (AUC) to analyze the performance of the developed models, and Bayesian information criterion (BIC) and Akaike Information Criterion (AIC) methods were used to analyze the relative goodness of fit of the models. We selected models with an 'excellent' grade of AUC value with 0.8 or more [29]. Models of clinical prediction formula with less BIC and AIC were considered more suitable models, but more weight was placed on the smaller BIC in order to reduce the enrolled risk factors as much as possible. Statistical analysis was conducted using SPSS version 22.0.0 (IBM Corp., Armonk, NY) and MedCalc version 12.5.0 (MedCalc Software bvba, Ostend, Belgium). A *p*-value of < 0.05 (two-sided) was considered statistically significant.

## Results

Based on the demographics of the participants, males were the predominant sex in the study population (2,802 men and 630 women). The mean participants age, body mass index (BMI), and Epworth Sleepiness Scale (ESS) score were 42.6 ± 13.5 years, 26.3 ± 4.1 kg/m^2^, and 10.5 ± 5.0, respectively. Moreover, the high-risk rate based on the Berlin questionnaire score was 76.9% (Table 1). Pearson’s correlation was used to analyze the correlation of AHI (a standard index for OSA diagnosis) or RDI (scored by adding RERA to the AHI) with the possible risk factors of demographic, anthropometric, sleep questionnaire, and physical exam factors. Many factors showed statistically significant correlations with AHI or RDI. Waist circumference, BMI, and neck circumference, which are factors related to body volume, showed sequentially high correlation with AHI or RDI, in that order. The enrolled participants were divided into the OSA group (having an AHI of ≥ 5) and non-OSA group (having an AHI of < 5). When looking at the correlation between possible risk factors and the presence or absence of OSA, waist circumference, age, and Berlin questionnaire showed sequentially high correlation (Table 2). Multicollinearity among the factors was considered by assessing the correlation among anthropometric factors, medical history, sleep questionnaire scores, and upper airway anatomical factors (S1 Table). This was to avoid falsely identifying variables as risk factors based on their association with true risk factors rather than that with the disease itself.

**Table 1:** Baseline demographic characteristics of the enrolled participants (n = 3,432).

Variables		Variables	
**Age (yr)**	42.6±13.5	**Hip circumference (cm)**	99.2±7.3
**Male:female ratio**	4.46:1	**PSQI score**	8.5±3.9
**HTN, n (%)**	864 (26.3)	**ESS score**	10.5±5.0
**DM, n (%)**	226 (6.9)	**Berlin questionnaire n (% of high risk group)**	2314(76.9)
**Allergy, n (%)**	114 (3.5)	**AHI**	24.1±24.3
**Height (m)**	1.70±0.09	**RDI**	31.3±24.2
**Weight (kg)**	76.2±14.7	**Tonsil grade (I/II/III/IV)**	972(51.5)/636(33.7)/200(10.6).11(.6)
**BMI (kg/m**^**2**^**)**	26.3±4.1	**Mallampati grade (I/II/III/IV)**	107(5.7)/364(19.3)/900(47.6)/518(27.4)
**Neck circumference (cm)**	37.1±3.6	**Uvula length (long/moderate/short)**	374(20.1)/1040(55.9)/448(24.1)
**Waist circumference (cm)**	91.8±10.4	**Oropharynx width (no/partial/complete obstruction)**	219(11.6)/1367(72.7)/294(15.6)

Mean ± standard deviation. BMI was calculated as weight in kilograms divided by height in meters squared (kg/m^2^). HTN = hypertension; DM = diabetes mellitus; BMI = body mass index; PSQI = Pittsburgh Sleep Quality Index; ESS = Epworth Sleepiness Scale; AHI = apnea-hypopnea index; RDI = respiratory distress index. Tonsil grade according to Friedman staging; tongue position according to modified Mallampati grading; uvula length categorized as long, moderate, and short; oropharyngeal width categorized as no, partial, and complete obstruction.

**Table 2:** Correlation analysis between the demographic factors and AHI or RDI.

Variables	AHI		RDI		OSA (0 = non-OSA, 1 = OSA)	
	coefficient	p	coefficient	p	coefficient	p
**Age (yr)**	.229	< .001	.260	< .001	.354	< .001
**Male:female ratio** (female = 0, male = 1)	.180	< .001	.197	< .001	.190	< .001
**HTN, n (%)**	.309	< .001	.313	< .001	.241	< .001
**DM, n (%)**	.139	< .001	.125	< .001	.117	< .001
Allergy, n (%)	-.045	< .001	-.041	< .001	.009	.690
Height (m)	.071	< .001	.067	< .001	.016	.342
**Weight (kg)**	.411	< .001	.394	< .001	.253	< .001
**BMI (kg/m**^**2**^**)**	.460	< .001	.444	< .001	.303	< .001
**Neck circumference (cm)**	.434	< .001	.438	< .001	.311	< .001
**Waist circumference (cm)**	.510	< .001	.502	< .001	.363	< .001
**Hip circumference (cm)**	.353	< .001	.333	< .001	.196	< .001
PSQI score	.015	.529	.013	.579	-.013	.583
**ESS score**	.101	< .001	.099	< .001	.047	.010
**Berlin questionnaire** (L = 0, H = 1)	.309	< .001	.347	< .001	.344	< .001
**Tonsil grade** (I/II/III/IV)	.113	< .001	.103	< .001	.096	.020
**Mallampati grade** (I/II/III/IV)	.228	< .001	.239	< .001	.248	< .001
**Uvula length** (long/moderate/short)	-.124	< .001	-.119	< .001	-.104	.001
**Oropharynx width** (no/partial/complete obstruction)	.200	< .001	.205	< .001	.191	< .001

Mean ± standard deviation. Pearson, Biserial, Rank Biserial, Point Biserial, and Phi correlation analysis. **Bold** means statistically significant. Statistical significance at *p* < 0.05 (two-sided). BMI was calculated as weight in kilograms divided by height in meters squared (kg/m^2^). AHI = apnea-hypopnea index; RDI = respiratory distress index; HTN = hypertension; DM = diabetes mellitus; BMI = body mass index; PSQI = Pittsburgh Sleep Quality Index; ESS = Epworth Sleepiness Scale. Tonsil grade according to Friedman staging; tongue position according to modified Mallampati staging; uvula length categorized as long, moderate, and short; oropharyngeal width categorized as no, partial, and complete obstruction.

Similarly, to analyze possible risk factors in other statistical methods, and to determine the possible risk factors (predictors) to be included in the clinical prediction formula, univariate regression analysis was used to analyze the impact of each possible predictor on OSA. The possible risk factors, after adjusting age and sex, demographic factors of HTN and DM, including body volume-related anthropometric factors of neck circumference, BMI, waist circumference, hip circumference, and weight, sleep questionnaire factors of ESS and Berlin, and upper airway obstruction-related anatomical factors of the tonsil, tongue, and oropharynx complete obstruction, were significantly associated with OSA (Table 3).

**Table 3:** Univariate regression analysis for predicting OSA (adjusted age and sex).

Variables	OR	95% LCI	95% UCI	p-value
**HTN**	3.050	2.210	4.211	< .001
**DM**	3.411	1.864	6.241	< .001
Allergy	.745	.452	1.230	.250
Height	0.598	.126	2.819	.515
**Weight**	1.052	1.042	1.061	< .001
**BMI**	1.212	1.176	1.249	< .001
**Obesity (BMI>25)**	2.983	2.439	3.648	< .001
**Neck circumference**	1.280	1.225	1.336	< .001
**Waist circumference**	1.077	1.064	1.090	< .001
**Hip circumference**	1.077	1.061	1.094	< .001
PSQI	1.011	.963	1.062	.655
**ESS**	1.026	1.004	1.048	.019
**Berlin**	4.328	3.415	5.484	< .001
**Tonsil**	1.720	1.355	2.183	< .001
**Tongue**	1.247	1.043	1.490	.015
Uvula (Short vs Long)	1.598	.977	2.612	.062
Uvula (Moderate vs Long)	.941	.605	1.463	.788
**Oropharynx (Complete vs No)**	.287	.155	.531	< .001
Oropharynx (Partial vs No)	.733	.476	1.128	.157

**Bold** means statistically significant. Statistical significance at *p* < 0.05 (two-sided). BMI was calculated as weight in kilograms divided by height in meters squared (kg/m^2^). HTN = hypertension; DM = diabetes mellitus; BMI = body mass index; PSQI = Pittsburgh Sleep Quality Index; ESS = Epworth Sleepiness Scale. Tonsil grade according to Friedman staging; tongue position according to modified Mallampati staging; uvula length categorized as long, moderate, and short; oropharyngeal width categorized as no, partial, and complete obstruction.

**Fig 2:**
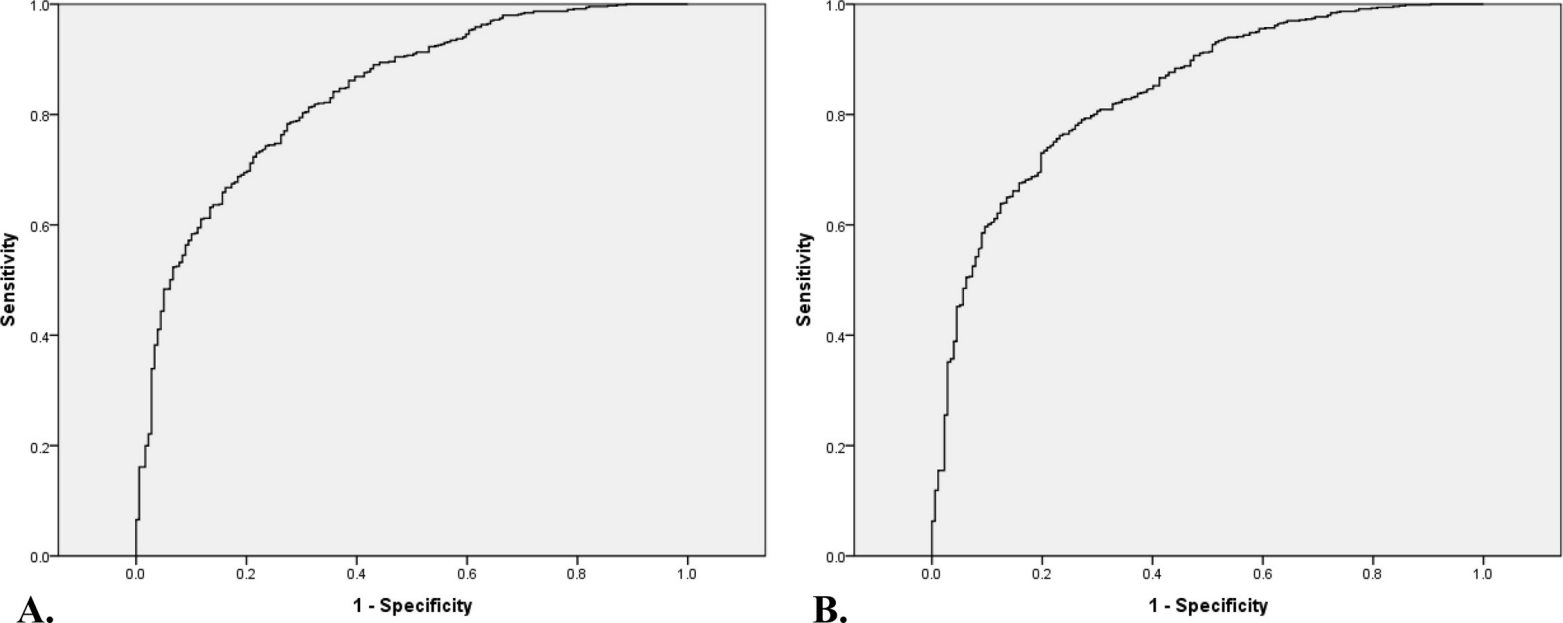
Receiver operating characteristic curve for the prediction formula for obstructive sleep apnea verified in the validation group (n = 916). A. Age, sex, BMI, HTN, Berlin questionnaire score, and tonsil grade. AUC = 0.835. B. Age, sex, BMI, NC, HTN, DM, ESS, and Berlin questionnaire score. AUC = 0.839. BMI, body mass index; NC, neck circumference; HTN, hypertension; DM, diabetes mellitus; ESS, Epworth sleepiness scale; Berlin, Berlin questionnaire; AUC, area under the curve.

Various clinical formula models were developed using a combination of significant possible risk factors based on the results of the correlation analysis and univariate analysis as well as previous clinical study results. Significant prediction factors were considered as those with a high correlation value, high odds-ratio value for univariate regression, and significant p value. Further, significant variables in the correlation analysis and univariate regression were excluded from our model if they were clinically judged as incompatible with our hypothesis. These clinical formulas were analyzed and developed using multivariate logistic regression with respect to the combination of the various predictors and the extent of their effect on OSA. And among them, 13 formulas with an AUC value of 0.8 or higher, were verified, graded as excellent, and selected using a validation set (n = 916) and by determining their sensitivities, specificities, and area under the ROC curve (AUC) values (Table 4, Fig 2 and S1 Fig). Moreover, the results of each multivariate regression analysis were validated using the Cox and Snell R^2^ and Nagelkerke R^2^ (Table 5 and S2 Table). The multivariate logistic regression of all selected equations showed that the Hosmer–Lemeshow value was greater than 0.05, indicating that the models of logistic regression were enough fit and, therefore, suitable.

**Table 4:** Possible risk factors and analyzed formula models.

	**Formulas with analyzed predictors for obstructive sleep apnea**	**Sensitivity**	**Specificity**	**Area under the curve**	**BIC**	**AIC**
Physical exam included	Age, Sex, BMI, NC, HTN, DM, ESS, Berlin, Tonsil, Tongue	0.764	0.767	0.831	685.131	633.342
	Age, Sex, BMI, HTN, ESS, Berlin, Tonsil, Tongue	0.754	0.768	0.831	693.397	650.936
	Age, Sex, BMI, HTN, Berlin, Tonsil, Tongue	0.769	0.768	0.833	689.075	651.333
	**Age, Sex, BMI, HTN, Berlin, Tonsil**	**0.776**	**0.757**	**0.835**	**682.906**	**649.881**
	Age, Sex, BMI, HTN, Berlin, Tongue	0.741	0.774	0.837	694.459	661.418
	Age, Sex, BMI, Berlin, Tonsil, Tongue	0.774	0.761	0.836	792.051	758.107
	Age, Sex, BMI, Berlin, Tonsil	0.771	0.761	0.836	785.210	756.116
	Age, Sex, BMI, Berlin, Tongue	0.830	0.689	0.839	798.129	769.022
Physical exam not-included	**Age, Sex, BMI, NC, HTN, DM, ESS, Berlin**	**0.749**	**0.770**	**0.839**	**1769.866**	**1719.530**
	Age, Sex, BMI, HTN, DM, ESS, Berlin	0.730	0.788	0.838	1784.049	1739.258
	Age, Sex, BMI, HTN, ESS, Berlin	0.730	0.782	0.838	1787.310	1748.104
	Age, Sex, BMI, HTN, Berlin	0.781	0.732	0.839	1780.632	1747.023
	Age, Sex, BMI, Berlin	0.769	0.753	0.840	1893.120	1864.822

BMI = body mass index; HTN = hypertension; DM = diabetes mellitus; ESS = Epworth Sleepiness Scale; Berlin = Berlin questionnaire. Tonsil grade according to Friedman staging; tongue position according to modified Mallampati staging; uvula length categorized as long, moderate, and short; oropharyngeal width categorized as no, partial, and complete obstruction.

**Table 5:** Multivariate logistic regression analysis for predicting OSA and validation of the regression analysis results and clinical formula for OSA with or without physical examination factors. A. Age, sex, BMI, HTN, Berlin questionnaire score, and tonsil grade. B. Age, sex, BMI, HTN, and Berlin questionnaire score.

<*A*. *Clinical formula with physical examination factors*>				
**Enrolled factors**	**A. Age, Sex, BMI, HTN, Berlin, Tonsil**			
**Clinical formula**	exp(−9.713+0.089*Age+1.095*Sex+0.360*HTN+0.127*BMI+1.471*Berlin+0.497*Tonsilgrade)/1+exp(−9.713+0.089*Age+1.095*Sex+0.360*HTN+0.127*BMI+1.471*Berlin+0.497*Tonsilgrade)			
**Variables**	**OR**	**95% LCI**	**95% UCI**	**p-value**
Constant	-9.713	0.000	0.924	< .001
Age	0.089	1.093	0.009	< .001
Sex	1.095	2.990	0.261	< .001
BMI	0.127	1.135	0.030	< .001
HTN	0.360	1.433	0.348	0.301
Berlin	1.471	4.352	0.216	< .001
Tonsil	0.497	1.644	0.149	< .001
**Measures of Fit for Logistic Regression**	Cox & Snell R square: 0.331			
	Nagelkerke R square: 0.480			
Statistical significance at p < 0.05 (two-sided). Tonsil grade according to Friedman staging. BMI = body mass index; HTN = hypertension; Berlin = Berlin questionnaire				
<*B*. *Clinical formula without physical examination factors*>				
**Enrolled factors**	**B. Age, Sex, BMI, Neck circumference, HTN, DM, ESS, Berlin,**			
Clinical formula	exp(−10.581+0.069*Age+0.610*Sex+0.572*HTN+0.520*DM+0.097*BMI+0.108*Neck+(−0.009*ESS)+1.102*Berlin)/1+exp(−10.581+0.069*Age+0.610*Sex+0.572*HTN+0.520*DM+0.097*BMI+0.108*Neck+(−0.009*ESS)+1.102*Berlin)			
**Variables**	**OR**	**95% LCI**	**95% UCI**	**p-value**
Constant	.000			< .001
Age	1.071	1.060	1.082	< .001
Sex	1.841	1.199	2.828	.005
BMI	1.102	1.053	1.152	< .001
Neck circumference	1.114	1.043	1.191	.001
HTN	1.771	1.188	2.640	.005
DM	1.682	.826	3.427	.152
ESS	.991	.967	1.016	.489
Berlin	3.010	2.302	3.936	< .001
**Measures of Fit for Logistic Regression**	Cox & Snell R square: 0.277			
	Nagelkerke R square: 0.400			
Statistical significance at *p* < 0.05 (two-sided)				
BMI = body mass index; HTN = hypertension; DM = diabetes mellitus; ESS = Epworth sleepiness scale; Berlin = Berlin questionnaire				

These 13 formulas were divided depending on whether physical examinations were required or not. In addition, the goodness of fit model was analyzed by BIC and AIC values, and the formulas showing as lower values of BIC and AIC as possible was selected. For ease of use, in order to include as few factors as possible in the formula, the BIC value was given more weight than AIC value was (Table 5). Among the developed formulas, the combination of “age, sex, HTN, BMI, Berlin questionnaire score, and tonsil grade” showed an excellent AUC value, a sensitivity, a specificity, the lowest BIC value, and an AIC value of 0.835, 0.776, 0.757, 682.906, and 649.881, respectively. The formula for this combination was as follows:

1 The probability of OSA (including physical examination)
  : exp(9.460 + 0.080*Age + 1.123*Sex + 0.316*HTN + 0.154*BMI + 1.277*Berlin + 0.300*Tonsil grade) / [1+exp(-9.460 + 0.080*Age + 1.123*Sex + 0.316*HTN + 0.154*BMI + 1.277*Berlin + 0.300*Tonsil grade)]

Among the formulas that did not include physical examination findings, the combination of “age, sex, HTN, DM, BMI, neck circumference, ESS, and Berlin questionnaire score” showed an excellent AUC value, a sensitivity, a specificity, the lowest BIC value, and an AIC value of 0.839, 0.749, 0.770, 1769.866, and 1719.530, respectively. The formula for this combination was as follows:

2 The probability of OSA (not-including physical examination)
  ∶ exp(-10.581 + 0.069*Age + 0.610*Sex + 0.572*HTN + 0.520*DM + 0.097*BMI + 0.108*NC + (-0.009*ESS) + 1.102*Berlin) / [1+exp(-10.581 + 0.069*Age + 0.610*Sex + 0.572*HTN + 0.520*DM + 0.097*BMI + 0.108*Neck + (-0.009*ESS) + 1.102*Berlin)]

## Discussion

Our study analyzed the risk factors for OSA and used them to develop clinical prediction formulas for efficient screening and diagnosis of patients with OSA naive to diagnostic tests due to problems regarding costs and medical resources; however, PSG remains essential for OSA diagnosis. We divided the enrolled participants into the development and validation cohorts. The development cohort was evaluated to identify risk factors for sleep apnea, which were used to develop our clinical prediction formulas. These developed clinical prediction formulas were verified in the validation group. To account for cases with and without a simple physical examination, we presented two formulas, i.e., one with and without the tonsil grade on physical examination. Both formulas showed appropriate sensitivity, specificity, and AUC values. Moreover, the clinical formula that included the physical examination results had slightly higher sensitivity and AUC values.

For example, applying the developed clinical prediction formulas to the enrolled participants:

1. If an age of 41 years, female sex, HTN, BMI of 34.7, neck circumference of 46.0, ESS of 10, Berlin high risk, and tonsil grade of 3 are included into the formula with the tonsil grade in the participants with study ID. 2,977:
  = exp(-9.460 + 0.080*Age + 1.123*Sex + 0.316*HTN + 0.154*BMI + 1.277*Berlin + 0.300*Tonsil grade) / [1 + exp(-9.460 + 0.080*Age + 1.123*Sex + 0.316*HTN + 0.154*BMI + 1.277*Berlin + 0.300*Tonsil grade)]
  = 0.982
  : There is a 98% risk of being diagnosed with sleep apnea.
2. If these parameters are adjusted into the formula without the tonsil grade:
  = exp(-10.581 + 0.069*Age + 0.610*Sex + 0.572*HTN + 0.520*DM + 0.097*BMI + 0.108*Neck + (-0.009*ESS) + 1.102*Berlin) / [1 + exp(-10.581 + 0.069*Age + 0.610*Sex + 0.572*HTN + 0.520*DM + 0.097*BMI + 0.108*Neck + (-.009*ESS) + 1.102*Berlin)]
  = 0.735
  : There is a 74% risk of being diagnosed with sleep apnea.
  : PSG of this specific particiapnts (study ID. 2,977) had severe sleep apnea with an AHI of 103.

The most crucial aspect of the development of the clinical prediction formulas for OSA was related to the risk factors for OSA, including age, sex, BMI, as well as a medical history of HTN and DM. However, this topic remains controversial. Specifically, OSA is known to affect uncontrolled HTN; however, it remains unclear how HTN affects the incidence or severity of OSA, as well as the risk factors for OSA. Some studies have reported HTN as a risk factor or predictor for OSA [30–32]; with certain medications for controlling HTN affecting OSA severity [33]. Further, the similarity of risk factors between HTN and OSA, including age, male sex, and obesity, may have influenced the analysis of HTN as a risk factor of OSA. Moreover, undiagnosed cardiac diseases, which may cause HTN and OSA, may have similar effects. Therefore, to select the appropriate clinical prediction formula, HTN should be included in the formula since it results in better sensitivity, specificity, and AUC values. And also we found that DM affected OSA. Previous studies have reported that DM affects HTN and, consequently, affects OSA [34]. Moreover, DM affects periodic breathing associated with OSA [35]. Further, type I DM affects OSA prevalence and severity [36]. However, we did not include it in the selected clinical prediction formula with physical exam since it showed worse sensitivity, specificity, and AUC values.

Among the anatomical factors, including those associated with the tonsil, tongue, uvula, and oropharynx, the tonsil and tongue grades affect OSA. The association of OSA with tonsil is dependent on the size. For simplicity and clarity, this was not considered a categorical variable and it was included in the formula as a continuous variable. Further, the inclusion of the tonsil grade alone, rather than both the tonsil and tongue grades, had better sensitivity and AUC values.

Rowley et al. [37] assessed 370 participants to determine the risk factors for OSA and develop clinical formulas. Their inclusion of various variables could have been advantageous; however, the cut-off AHI value of 10 or 20 was unusual. Moreover, they did not consider multicollinearity between each factor. Therefore, compared with our study, this previous study presented lower AUC values. Sahin et al. [38] included social-related factors, including alcohol drinking and smoking; however, their formula was not verified. Further, factors considered to affect each other, including BMI, waist circumference, and hip circumference, were included within the same formula. Kim et al. [39] subdivided the snoring factors; moreover, they included skull measurements, which was not included in any other studies. However, multicollinearity was not considered, validation was not performed, and relatively low AUC values were obtained. Additionally, generalizing these developed clinical formulas for OSA is controversial since they contain factors associated with radiologic examination, which must be performed in hospitals. Moreover, a small number of participants was included and verification using separate participants was not performed. In addition, various other formulas for predicting OSA have been developed so far [21, 40], such as a decision tree for OSA [41], a formula that allows perioperative patients to predict OSA [42], or a formula that can predict OSA in pediatric populations [43]. However, most of the clinical prediction formulas did not gain popularity after development, particularly because each formula came with their own set of parameters that were often population specific or many factors that need to be plugged into the formula were not available. Therefore, we have tried to present clinical formulas in which various variables are combined so that they can be adjusted according to various conditions. In addition, one of the two clinical formulas that are considered to be the most suitable models, based on various statistical verifications, does not require the tonsil size for its calculation, allowing for more flexibility.

There have been studies on the effects of nasal congestion, allergic rhinitis, and non-allergic rhinitis on sleep. Generally, stuffy nose or allergic/non-allergic rhinitis is expected to affect sleep to some extent; however, there have been controversial findings. Previous studies have reported that nasal congestion affects OSA severity [44]. Allergic rhinitis does not affect OSA; however, it affects habitual snoring [45]. Conversely, non-allergic rhinitis has been reported to affect OSA severity [46]. We did not include some factors, including nasal symptoms, non-allergic rhinitis, or snoring; furthermore, there was no significant relationship of allergic rhinitis with OSA.

The present study was based on a large number of participants and was conducted in a single institution at a single sleep research center. One strength of this study is that we performed a validation process suitable for generalizing the developed formulas by dividing the enrolled participants into the analysis and validation groups. Previous studies have reported risk factors for OSA, including age, sex, obesity, hormonal factors, ethnicity, smoking, upper-airway anatomical factors, and congenital craniofacial syndromes. In our study, we limited the race of our participants to Asians since OSA severity is affected by racial differences [47]. Contrastingly, the limited applicability of this formula to only Asians may be a limitation; however, the development of a more explicit formula by controlling for racial differences may be a strength. Further, among the previously reported formulas, our formula had the highest AUC value, which is a representative indicator of a formula’s suitability. However, we did not include well-known risk factors for OSA, including menopause, smoking [48], lifestyle, and occupational stress [49]. The inclusion of these risk factors could yield a more suitable formula.

### Conclusions

The various risk factors and predictors for OSA as well as our clinical prediction formula can be utilized for screening of Asian patients with OSA. These tools employ efficient methods to effectively utilize medical resources for obstructive sleep apnea screening.

## Supporting information

S1 Fig Receiver operating characteristic curve for the prediction formula for obstructive sleep apnea verified in the validation group (n = 916). A. Age, sex, BMI, NC, HTN, DM, ESS score, Berlin questionnaire score, tonsil grade, and tongue grade. B. Age, sex, BMI, HTN, ESS score, Berlin questionnaire score, tonsil grade, and tongue grade. C. Age, sex, BMI, HTN, Berlin questionnaire score, tonsil grade, and tongue grade. D. Age, sex, BMI, HTN, Berlin questionnaire score, and tongue grade. E. Age, sex, BMI, Berlin questionnaire score, tonsil grade, and tongue grade. F. Age, sex, BMI, Berlin questionnaire score, and tonsil grade. G. Age, sex, BMI, Berlin questionnaire score, and tongue grade. H. Age, sex, BMI, NC, HTN, DM, ESS score, and Berlin questionnaire score. I. Age, sex, BMI, HTN, DM, ESS score, and Berlin questionnaire score. J. Age, sex, BMI, HTN, ESS score, and Berlin questionnaire score. K. Age, sex, BMI, and Berlin questionnaire score. Abbreviations: BMI, body mass index; NC, neck circumference; HTN, hypertension; DM, diabetes mellitus; ESS, Epworth Sleepiness Scale.
(TIF)

S1 Table Correlation analysis among the enrolled factors for multicollinearity. (DOCX)

S2 Table Multivariate regression analysis for predicting OSA and validation of the regression analysis results and clinical formula for OSA with or without physical examination factors. (DOCX)
