# Supplementary figures and images for: Risk factors and clinical prediction formula for the evaluation of obstructive sleep apnea in Asian adults
Source: PLoS One. 2021 Feb 2;16(2):e0246399. doi: 10.1371/journal.pone.0246399 (PMC7853448; doi:10.1371/journal.pone.0246399)

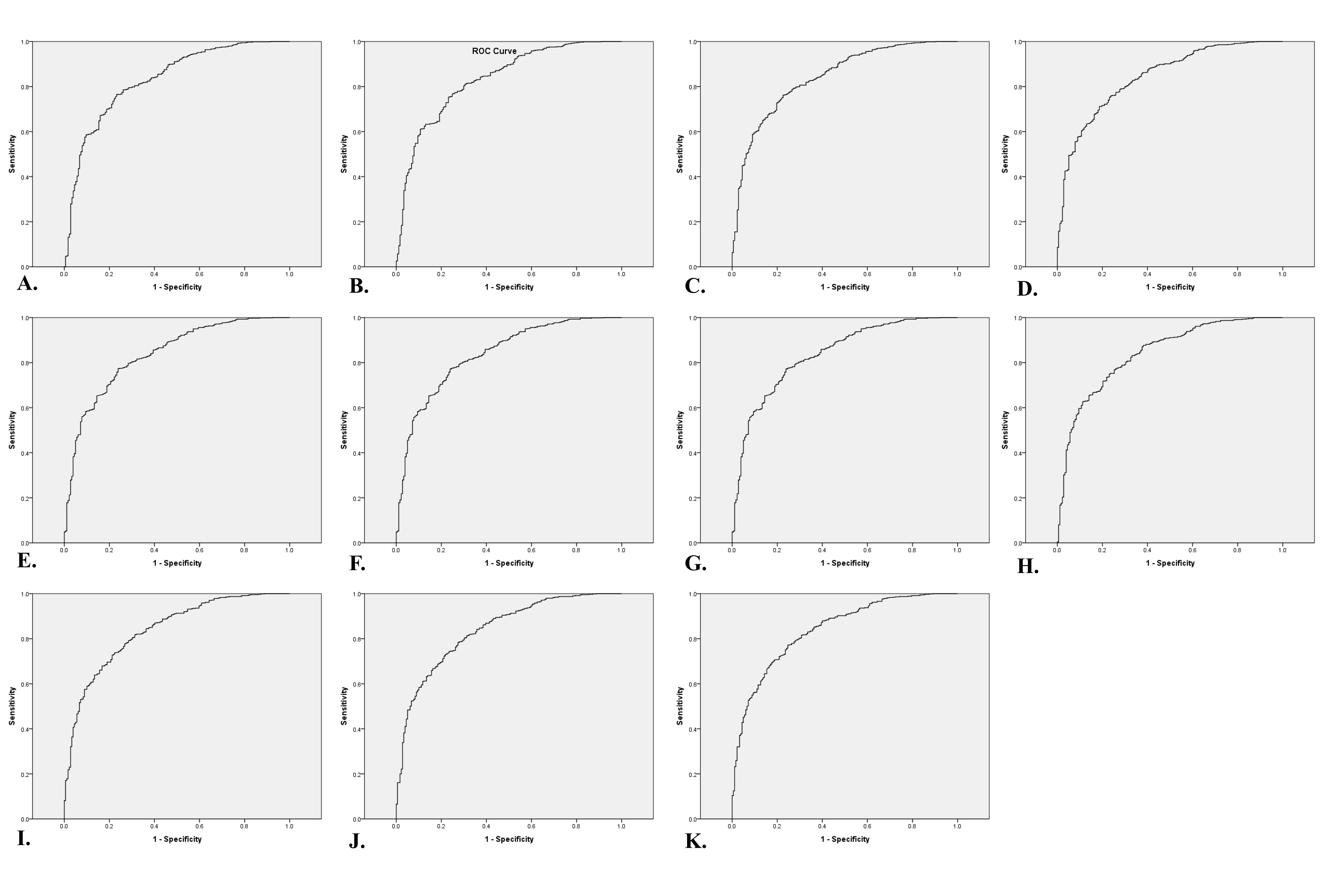

Supplement: S1 Fig — A. Age, sex, BMI, NC, HTN, DM, ESS score, Berlin questionnaire score, tonsil grade, and tongue grade. B. Age, sex, BMI, HTN, ESS score, Berlin questionnaire score, tonsil grade, and tongue grade. C. Age, sex, BMI, HTN, Berlin questionnaire score, tonsil grade, and tongue grade. D. Age, sex, BMI, HTN, Berlin questionnaire score, and tongue grade. E. Age, sex, BMI, Berlin questionnaire score, tonsil grade, and tongue grade. F. Age, sex, BMI, Berlin questionnaire score, and tonsil grade. G. Age, sex, BMI, Berlin questionnaire score, and tongue grade. H. Age, sex, BMI, NC, HTN, DM, ESS score, and Berlin questionnaire score. I. Age, sex, BMI, HTN, DM, ESS score, and Berlin questionnaire score. J. Age, sex, BMI, HTN, ESS score, and Berlin questionnaire score. K. Age, sex, BMI, and Berlin questionnaire score. Abbreviations: BMI, body mass index; NC, neck circumference; HTN, hypertension; DM, diabetes mellitus; ESS, Epworth Sleepiness Scale. (TIF) [file pone.0246399.s001.tif]
