# Supplementary material for: Risk factors and clinical prediction formula for the evaluation of obstructive sleep apnea in Asian adults
Source: PLoS One. 2021 Feb 2;16(2):e0246399. doi: 10.1371/journal.pone.0246399 (PMC7853448; doi:10.1371/journal.pone.0246399)
Supplement: S1 Table — (DOCX) [file pone.0246399.s002.docx]

**S1 Table. Correlation analysis among the enrolled factors for multicollinearity**

| Variables | HTN, n(%) | | DM, n(%) | |
| --- | --- | --- | --- | --- |
|  | coefficient | p | coefficient | p |
| HTN, n(%) | 1 |  | .274 | <.001 |
| DM, n(%) | .274 | <.001 | 1 |  |

Pearson’s correlation analysis. Statistical significance = *p*<0.05.

| Variables | Height, m | | | Weight, kg | | | BMI, kg/m^2^ | | | Neck circumference, cm | | | Waist circumference, cm | | | Hip circumference, cm | |
| --- | --- | --- | --- | --- | --- | --- | --- | --- | --- | --- | --- | --- | --- | --- | --- | --- | --- |
|  | r | p | r | | p | r | | p | r | | p | r | | p | r | | p |
| Height, m | 1 |  | .020 | | .304 | .001 | | .941 | .011 | | .599 | .017 | | .395 | .023 | | .260 |
| Weight, kg | .020 | .304 | 1 | |  | .843 | | <.001 | .783 | | <.001 | .805 | | <.001 | .830 | | <.001 |
| BMI, kg/m^2^ | .001 | .941 | .843 | | <.001 | 1 | |  | .629 | | <.001 | .842 | | <.001 | .833 | | <.001 |
| Neck circumference, cm | .011 | .599 | .783 | | <.001 | .629 | | <.001 | 1 | |  | .653 | | <.001 | .578 | | <.001 |
| Waist circumference, cm | .017 | .395 | .805 | | <.001 | .842 | | <.001 | .653 | | <.001 | 1 | |  | .808 | | <.001 |
| Hip circumference, cm | .023 | .260 | .830 | | <.001 | .833 | | <.001 | .578 | | <.001 | .808 | | <.001 | 1 | |  |

Pearson’s correlation analysis. Statistical significance = *p*<0.05.

| Variables | ESS score | | Berlin questionnaire (L=0, H=1) | | PSQI score | |
| --- | --- | --- | --- | --- | --- | --- |
|  | r | p | r | p | r | p |
| ESS score | 1 |  | .228 | <.001 | .184 | <.001 |
| Berlin questionnaire (L=0, H=1) | .228 | <.001 | 1 |  | .189 |  |
| PSQI score | .184 | <.001 | .189 | <.001 | 1 | <.001 |

Pearson’s correlation analysis. Statistical significance = *p*<0.05.

| Variables | Tonsil grade (I/II/III/IV) | | Tongue position (I/II/III/IV) | | Uvula length (long/moderate/short) | | Oropharynx width (wide/moderate/narrow) | |
| --- | --- | --- | --- | --- | --- | --- | --- | --- |
|  | r | p | r | p | r | p | r | p |
| Tonsil grade (I/II/III/IV) | 1 |  | .060 | .061 | -.228 | <.001 | .376 | <.001 |
| Tongue position (I/II/III/IV) | .060 | .061 | 1 |  | -.184 | <.001 | .319 | <.001 |
| Uvula length (long/moderate/short) | -.228 | <.001 | -.184 | <.001 | 1 |  | -.306 | <.001 |
| Oropharynx width (wide/moderate/narrow) | .376 | <.001 | .319 | <.001 | -.306 | <.001 | 1 |  |

Pearson’s correlation analysis. Statistical significance = *p*<0.05.
