# Supplementary material for: Risk factors and clinical prediction formula for the evaluation of obstructive sleep apnea in Asian adults
Source: PLoS One. 2021 Feb 2;16(2):e0246399. doi: 10.1371/journal.pone.0246399 (PMC7853448; doi:10.1371/journal.pone.0246399)
Supplement: S2 Table — (DOCX) [file pone.0246399.s003.docx]

**S2 Table. Multivariate regression analysis for predicting OSA and validation of the regression analysis results and clinical formula for OSA with or without physical examination factors.**

A. Age, Sex, HTN, DM, BMI, neck circumference, ESS score, Berlin questionnaire score, tonsil grade, and tongue grade. B. Age, sex, HTN, BMI, ESS score, Berlin questionnaire score, tonsil grade, and tongue grade. C. Age, sex, HTN, BMI, Berlin questionnaire score, tonsil grade, and tongue grade. D. Age, sex, HTN, BMI, Berlin questionnaire score, and tongue grade. E. Age, sex, BMI, Berlin questionnaire score, tonsil grade, and tongue grade. F. Age, sex, BMI, Berlin questionnaire score, and tonsil grade. G. Age, sex, BMI, Berlin questionnaire score, and tongue grade. H. Age, sex, HTN, DM, BMI, ESS score, and Berlin questionnaire score. I. Age, sex, HTN, BMI, ESS score, and Berlin questionnaire score. J. Age, sex, BMI, HTN, and Berlin questionnaire score. K. Age, sex, BMI, and Berlin questionnaire score

OSA = obstructive sleep apnea; BMI = body mass index; HTN = hypertension; DM = diabetes mellitus; ESS = Epworth Sleepiness Scale

<with physical exam>

| **Enrolled factors** | **A. Age, Sex, HTN, DM, BMI, Neck circumference, ESS, Berlin, Tonsil, Tongue** | | | | | |
| --- | --- | --- | --- | --- | --- | --- |
| **Clinical formula** | $\boldsymbol{exp}\boldsymbol{(-13.524+0.092*Age+0.265*Sex+0.230*HTN+0.938*DM+0.052*BMI+0.187*Neck+(-0.034*ESS)+1.579*Berlin+0.514*Tonsil grade+(-0.143*Tongue grade))/1+}\boldsymbol{exp}\boldsymbol{(-13.524+0.092*Age+0.265*Sex+0.230*HTN+0.938*DM+0.052*BMI+0.187*Neck+(-0.034*ESS)+1.579*Berlin+0.514*Tonsil grade+(-0.143*Tongue grade))}$ | | | | | |
| **Variables** | **Estimate** | **OR** | **Standard error** | **p-value** | **95% LCI** | **95% UCI** |
| Constant | -13.524 | <.001 | 1.651 | <.001 |  |  |
| Age | .092 | 1.097 | .009 | <.001 | 1.077 | 1.117 |
| Sex | .265 | 1.303 | .386 | .493 | .612 | 2.775 |
| HTN | .230 | 1.259 | .354 | .516 | .629 | 2.522 |
| DM | .938 | 2.554 | .782 | .230 | .552 | 11.816 |
| BMI | .052 | 1.054 | .039 | .182 | .976 | 1.138 |
| Neck circumference | .187 | 1.206 | .060 | .002 | 1.071 | 1.357 |
| ESS | -.034 | .967 | .021 | .105 | .928 | 1.007 |
| Berlin | 1.579 | 4.851 | .230 | <.001 | 3.093 | 7.607 |
| Tonsil | .514 | 1.673 | .153 | .001 | 1.240 | 2.255 |
| Tongue | -.143 | .867 | .115 | .216 | .692 | 1.087 |
| **Measures of Fit for Logistic Regression** | Cox & Snell R square: 0.344  Nagelkerke R square: 0.500 | | | | | |

| **Enrolled factors** | **B. Age, Sex, HTN, BMI, ESS, Berlin, Tonsil, Tongue** | | | | | |
| --- | --- | --- | --- | --- | --- | --- |
| **Clinical formula** | $\boldsymbol{exp}\boldsymbol{(-9.559+0.091*Age+1.106*Sex+0.330*HTN+0.132*BMI+(-0.032*ESS)+1.572*Berlin+0.511*Tonsil grade+(-0.081*Tongue grade))/1+}\boldsymbol{exp}\boldsymbol{(-9.559+0.091*Age+1.106*Sex+0.330*HTN+0.132*BMI+(-0.032*ESS)+1.572*Berlin+0.511*Tonsil grade+(-0.081*Tongue grade))}$ | | | | | |
| **Variables** | **Estimate** | **OR** | **Standard error** | **p-value** | **95% LCI** | **95% UCI** |
| Constant | -9.559 | <.001 | .930 | <.001 |  |  |
| Age | .091 | 1.095 | .009 | <.001 | 1.076 | 1.115 |
| Sex | 1.106 | 3.021 | .261 | <.001 | 1.810 | 5.042 |
| HTN | .330 | 1.391 | .349 | .345 | .701 | 2.759 |
| BMI | .132 | 1.141 | .030 | <.001 | 1.076 | 1.211 |
| ESS | -.032 | .969 | .021 | .122 | .931 | 1.008 |
| Berlin | 1.572 | 4.815 | .226 | <.001 | 3.094 | 7.493 |
| Tonsil | .511 | 1.667 | .151 | .001 | 1.241 | 2.239 |
| Tongue | -.081 | .922 | .111 | .464 | .742 | 1.146 |
| **Measures of Fit for Logistic Regression** | Cox & Snell R square: 0.333  Nagelkerke R square: 0.483 | | | | | |

| **Enrolled factors** | **C. Age, Sex, HTN, BMI, Berlin, Tonsil, Tongue** | | | | | |
| --- | --- | --- | --- | --- | --- | --- |
| **Clinical formula** | $\boldsymbol{exp}\boldsymbol{(-9.445+0.081*Age+1.123*Sex+0.318*HTN+0.155*BMI+1.280*Berlin+0.300*Tonsil grade+(-0.014*Tongue grade))/1+}\boldsymbol{exp}\boldsymbol{(-9.445+0.081*Age+1.123*Sex+0.318*HTN+0.155*BMI+1.280*Berlin+0.300*Tonsil grade+(-0.014*Tongue grade))}$ | | | | | |
| **Variables** | **Estimate** | **OR** | **Standard error** | **p-value** | **95% LCI** | **95% UCI** |
| Constant | -9.445 | <.001 | .663 | <.001 |  |  |
| Age | .081 | 1.084 | .006 | <.001 | 1.071 | 1.097 |
| Sex | 1.123 | 3.073 | .182 | <.001 | 2.151 | 4.389 |
| HTN | .318 | 1.374 | .230 | .168 | .875 | 2.157 |
| BMI | .155 | 1.167 | .021 | <.001 | 1.120 | 1.217 |
| Berlin | 1.280 | 3.596 | .158 | <.001 | 2.638 | 4.902 |
| Tonsil | .300 | 1.350 | .098 | .002 | 1.113 | 1.638 |
| Tongue | -.014 | .986 | .086 | .868 | .833 | 1.166 |
| **Measures of Fit for Logistic Regression** | Cox & Snell R square: 0.290  Nagelkerke R square: 0.436 | | | | | |

| **Enrolled factors** | **D. Age, Sex, HTN, BMI, Berlin, Tongue** | | | | | |
| --- | --- | --- | --- | --- | --- | --- |
| **Clinical formula** | $\boldsymbol{exp}\boldsymbol{(-9.071+0.087*Age+1.135*Sex+0.373*HTN+0.138*BMI+1.475*Berlin+(-0.050*Tongue grade))/1+}\boldsymbol{exp}\boldsymbol{(-9.071+0.087*Age+1.135*Sex+0.373*HTN+0.138*BMI+1.475*Berlin+(-0.050*Tongue grade))}$ | | | | | |
| **Variables** | **Estimate** | **OR** | **Standard error** | **p-value** | **95% LCI** | **95% UCI** |
| Constant | -9.071 | 0<.001 | 0.891 | < .001 |  |  |
| Age | 0.087 | 1.091 | 0.009 | < .001 | 1.072 | 1.110 |
| Sex | 1.135 | 3.110 | 0.259 | < .001 | 1.871 | 5.168 |
| HTN | 0.373 | 1.451 | 0.347 | 0.283 | 0.735 | 2.867 |
| BMI | 0.138 | 1.148 | 0.029 | < .001 | 1.084 | 1.216 |
| Berlin | 1.475 | 4.370 | 0.216 | < .001 | 2.863 | 6.669 |
| Tongue | -0.050 | 0.952 | 0.110 | 0.653 | 0.767 | 1.181 |
| **Measures of Fit for Logistic Regression** | Cox & Snell R square: 0.322  Nagelkerke R square: 0.467 | | | | | |

| **Enrolled factors** | **E. Age, Sex, BMI, Berlin, Tonsil, Tongue** | | | | | |
| --- | --- | --- | --- | --- | --- | --- |
| **Clinical formula** | $\boldsymbol{exp}\boldsymbol{(-9.828+0.086*Age+1.014*Sex+0.140*BMI+1.484*Berlin+0.486*Tonsil grade+(-0.010*Tongue grade))/1+}\boldsymbol{exp}\boldsymbol{(-9.828+0.086*Age+1.014*Sex+0.140*BMI+1.484*Berlin+0.486*Tonsil grade+(-0.010*Tongue grade))}$ | | | | | |
| **Variables** | **Estimate** | **OR** | **Standard error** | **p-value** | **95% LCI** | **95% UCI** |
| Constant | -9.828 | <.001 | .833 | <.001 |  |  |
| Age | .086 | 1.090 | .008 | <.001 | 1.073 | 1.108 |
| Sex | 1.014 | 2.757 | .242 | <.001 | 1.717 | 4.427 |
| BMI | .140 | 1.150 | .027 | <.001 | 1.090 | 1.214 |
| Berlin | 1.484 | 4.410 | .199 | <.001 | 2.986 | 6.513 |
| Tonsil | .486 | 1.626 | .139 | <.001 | 1.239 | 2.134 |
| Tongue | -.010 | .990 | .103 | .926 | .809 | 1.213 |
| **Measures of Fit for Logistic Regression** | Cox & Snell R square: 0.322  Nagelkerke R square: 0.465 | | | | | |

| **Enrolled factors** | **F. Age, Sex, BMI, Berlin, Tonsil** | | | | | |
| --- | --- | --- | --- | --- | --- | --- |
| **Clinical formula** | $\boldsymbol{exp}\boldsymbol{(-9.837+0.086*Age+1.014*Sex+0.140*BMI+1.482*Berlin+0.485*Tonsil grade)/1+}\boldsymbol{exp}\boldsymbol{(-9.837+0.086*Age+1.014*Sex+0.140*BMI+1.482*Berlin+0.485*Tonsil grade)}$ | | | | | |
| **Variables** | **Estimate** | **OR** | **Standard error** | **p-value** | **95% LCI** | **95% UCI** |
| Constant | -9.837 | <.001 | .827 | <.001 |  |  |
| Age | .086 | 1.090 | .008 | <.001 | 1.073 | 1.107 |
| Sex | 1.014 | 2.756 | .242 | <.001 | 1.717 | 4.426 |
| BMI | .140 | 1.150 | .027 | <.001 | 1.090 | 1.213 |
| Berlin | 1.482 | 4.401 | .198 | <.001 | 2.987 | 6.483 |
| Tonsil | .485 | 1.624 | .139 | <.001 | 1.238 | 2.131 |
| **Measures of Fit for Logistic Regression** | Cox & Snell R square: 0.322  Nagelkerke R square: 0.465 | | | | | |

| **Enrolled factors** | **G. Age, Sex, BMI, Berlin, Tongue** | | | | | |
| --- | --- | --- | --- | --- | --- | --- |
| **Clinical formula** | $\boldsymbol{exp}\boldsymbol{(-9.248+0.083*Age+1.055*Sex+0.147*BMI+1.467*Berlin+0.015*Tongue grade)/1+}\boldsymbol{exp}\boldsymbol{(-9.248+0.083*Age+1.055*Sex+0.147*BMI+1.467*Berlin+0.015*Tongue grade)}$ | | | | | |
| **Variables** | **Estimate** | **OR** | **Standard error** | **p-value** | **95% LCI** | **95% UCI** |
| Constant | -9.248 | <.001 | .799 | <.001 |  |  |
| Age | .083 | 1.087 | .008 | <.001 | 1.070 | 1.104 |
| Sex | 1.055 | 2.871 | .240 | <.001 | 1.794 | 4.596 |
| BMI | .147 | 1.159 | .027 | <.001 | 1.099 | 1.222 |
| berlin | 1.467 | 4.335 | .197 | <.001 | 2.948 | 6.373 |
| Tongue | .015 | 1.015 | .103 | .882 | .830 | 1.243 |
| **Measures of Fit for Logistic Regression** | Cox & Snell R square: 0.313  Nagelkerke R square: 0.453 | | | | | |

<without physical exam>

| **Enrolled factors** | **H. Age, Sex, HTN, DM, BMI, ESS, Berlin,** | | | | | |
| --- | --- | --- | --- | --- | --- | --- |
| **Clinical formula** | $\boldsymbol{exp}\boldsymbol{(-8.293+0.068*Age+1.120*Sex+0.597*HTN+0.606*DM+0.144*BMI+(-0.008*ESS)+1.122*Berlin)/1+}\boldsymbol{exp}\boldsymbol{(-8.293+0.068*Age+1.120*Sex+0.597*HTN+0.606*DM+0.144*BMI+(-0.008*ESS)+1.122*Berlin)}$ | | | | | |
| **Variables** | **Estimate** | **OR** | **Standard error** | **p-value** | **95% LCI** | **95% UCI** |
| Constant | -8.293 | <.001 | .553 | <.001 |  |  |
| Age | .068 | 1.071 | .005 | <.001 | 1.060 | 1.082 |
| Sex | 1.120 | 3.066 | .154 | <.001 | 2.269 | 4.144 |
| HTN | .597 | 1.816 | .203 | .003 | 1.220 | 2.704 |
| DM | .606 | 1.834 | .364 | .096 | .899 | 3.742 |
| BMI | .144 | 1.155 | .018 | <.001 | 1.116 | 1.196 |
| ESS | -.008 | .992 | .013 | .507 | .968 | 1.016 |
| Berlin | 1.122 | 3.072 | .136 | <.001 | 2.353 | 4.010 |
| **Measures of Fit for Logistic Regression** | Cox & Snell R square: 0.274  Nagelkerke R square: 0.395 | | | | | |

| **Enrolled factors** | **I. Age, Sex, HTN, BMI, ESS, Berlin,** | | | | | |
| --- | --- | --- | --- | --- | --- | --- |
| **Clinical formula** | $\boldsymbol{exp}\boldsymbol{(-8.304+0.070*Age+1.106*Sex+0.655*HTN+0.143*BMI+(-0.010*ESS)+1.138*Berlin)/1+}\boldsymbol{exp}\boldsymbol{(-8.304+0.070*Age+1.106*Sex+0.655*HTN+0.143*BMI+(-0.010*ESS)+1.138*Berlin)}$ | | | | | |
| **Variables** | **Estimate** | **OR** | **Standard error** | **p-value** | **95% LCI** | **95% UCI** |
| Constant | -8.304 | <.001 | .551 | <.001 |  |  |
| Age | .070 | 1.072 | .005 | <.001 | 1.061 | 1.083 |
| Sex | 1.106 | 3.022 | .153 | <.001 | 2.241 | 4.077 |
| HTN | .655 | 1.924 | .201 | .001 | 1.298 | 2.854 |
| BMI | .143 | 1.154 | .018 | <.001 | 1.115 | 1.195 |
| ESS | -.010 | .990 | .012 | .410 | .966 | 1.014 |
| Berlin | 1.138 | 3.120 | .135 | <.001 | 2.393 | 4.069 |
| **Measures of Fit for Logistic Regression** | Cox & Snell R square: 0.273  Nagelkerke R square: 0.393 | | | | | |

| **Enrolled factors** | **J. Age, Sex, BMI, HTN, Berlin** | | | | | |
| --- | --- | --- | --- | --- | --- | --- |
| **Clinical formula** | $\boldsymbol{exp(-8.480+0.070*Age+1.121*Sex+0.569*HTN+0.152*BMI+1.108*Berlin)/1+exp(-8.480+0.070*Age+1.121*Sex+0.569*HTN+0.152*BMI+1.108*Berlin)}$ | | | | | |
| **Variables** | **Estimate** | **OR** | **Standard error** | **p-value** | **95% LCI** | **95% UCI** |
| Constant | -8.480 | <.001 |  | <.001 |  |  |
| Age | .070 | 1.072 | .004 | <.001 | 1.063 | 1.081 |
| Sex | 1.121 | 3.068 | .130 | <.001 | 2.376 | 3.961 |
| BMI | .569 | 1.164 | .168 | <.001 | 1.130 | 1.198 |
| HTN | .152 | 1.767 | .015 | .001 | 1.272 | 2.455 |
| Berlin | 1.108 | 3.030 | .113 | <.001 | 2.426 | 3.783 |
| **Measures of Fit for Logistic Regression** | Cox & Snell R square: 0.267  Nagelkerke R square: 0.393 | | | | | |

| **Enrolled factors** | **K. Age, Sex, BMI, Berlin,** | | | | | |
| --- | --- | --- | --- | --- | --- | --- |
| **Clinical formula** | $\boldsymbol{exp}\left( \boldsymbol{-8.842+0.074*Age+1.112*Sex+.155*BMI+1.18*Berlin} \right)\boldsymbol{/1+}\boldsymbol{exp}\boldsymbol{(-8.842+0.074*Age+1.112*Sex+.155*BMI+1.18*Berlin)}$ | | | | | |
| **Variables** | **Estimate** | **OR** | **Standard error** | **p-value** | **95% LCI** | **95% UCI** |
| Constant | -8.842 | <.001 | .518 | <.001 |  |  |
| Age | .074 | 1.076 | .005 | <.001 | 1.066 | 1.087 |
| Sex | 1.112 | 3.041 | .148 | <.001 | 2.274 | 4.068 |
| BMI | .155 | 1.167 | .017 | <.001 | 1.129 | 1.207 |
| Berlin | 1.189 | 3.283 | .126 | <.001 | 2.566 | 4.200 |
| **Measures of Fit for Logistic Regression** | Cox & Snell R square: 0.268  Nagelkerke R square: 0.386 | | | | | |
